# Description and management of *Aspergillus* section *Nigri* causing post-harvest bulbs rot of onion

**DOI:** 10.1038/s41598-024-53849-9

**Published:** 2024-03-13

**Authors:** Eman G. A. M. El-Dawy, Mohamed A. Hussein, Safaa El‑Nahas

**Affiliations:** 1 Botany and Microbiology Department, Faculty of Science, South Valley University, Qena, Egypt; 2 Applied and Environmental Microbiology Center, South Valley University, Qena, Egypt; 3 Chemistry Department, Faculty of Science, South Valley University, Qena, 83523 Egypt

**Keywords:** Onions, *Aspergillus* section *Nigri*, Eco-friendly, Pathogenicity, Biochemistry, Microbiology, Environmental sciences, Health care

## Abstract

When onions are improperly stored, a post-harvest disease known as black mold of onion bulbs can result in considerable economic losses. *Aspergillus* section *Nigri*, one of many species, has been implicated in the development of black mold. In the present study, rot onion bulbs were collected from markets in Qena, Egypt. Thirteen *Aspergillus* section *Nigri* isolates were obtained and identified by morphological and molecular characterization. The ochratoxins potential of isolated *A.* section *Nigri* was tested, and three isolates were producers at the range of 1.5–15 ppm. For the presence of *pks* gene, no amplification product was detected. Using the fungal growth inhibition test, the isolates of *A. niger* were inhibited by eco-friendly materials Cement and Zeolite. Cement exhibited maximum percentage growth inhibition against the tested isolates at 74.7–86.7%. The pathogenicity activity of the *A. niger* isolates was tested by inoculation of healthy onion bulbs, other onion bulbs covered with Cement and Zeolite before inoculation by *A. niger* was used. The two treatments significantly reduced bulbs rot disease of onion than untreated bulbs. Seven and nine isolates showed 0% rot on covered bulbs by Cement and Zeolite, respectively as compared with inoculated onions, which exhibited rot ranging from 55 to 80%. Using eco-friendly materials with efficiency against post-harvest bulbs rot of onion was evaluated in this study.

## Introduction

The ability of onion storage is greatly influenced by the environment and storage techniques^1^. Black mold disease cannot thrive in temperate climates due to low relative humidity and mild temperatures^2^. Keeping onions at room temperature for extended periods in the tropics is challenging due to the hot and humid weather^3^. Temperatures beyond 30 °C and relative humidity of 90–100% have been known to cause severe losses during storage. Significantly less pollution was produced at 20–25 °C temperatures and relative humidity of 70–80%^2,4^.

*Aspergillus niger* aggregate refers to ten taxa with remarkably similar morphologies: *A. costaricaensis*, *A. eucalypticola, A. luchuensis*, *A. neoniger*, *A. piperis*, *A. tubingensis* and *A. vadensis* (*A. tubingensis* clade), *A. niger* and *A. welwitschiae* (*A. niger* clade), and *A. brasiliensis* (*A. brasiliensis* clade), are generally referred as *Aspergillus niger* aggregate^5^.

The macro- and micro-morphological distinctions between several species of *Aspergillus* section *Nigri* are difficult to distinguish (black aspergilli). Molecular techniques have been crucial for differentiating these species^6^.

Worldwide research has been done on the important of this particular group of species as food product contaminants in terms of their prevalence and ability to produce mycotoxins. Ochratoxin A (OTA) and fumonisin B2 (FB2) are two mycotoxins frequently associated with *Aspergillus niger* aggregate. However, only fungal species of *A. niger* and *A. welwitschiae* are capable of producing these mycotoxins both in culture medium and on natural substrates^7^. *A. welwitschiae* is a species that was recently separated from the *A. niger* taxon^8^. No other *A.* *niger* aggregate species have been identified as OTA and/or FB2 producers. FB2 is neurotoxic and hepatotoxic to animals and has also been linked to esophageal tumors in humans; OTA is a nephrotoxic and carcinogenic mycotoxin^9^.

Cement manufacturing is one of Egypt's most vital sectors. This industry is intertwined with the construction and building industries and infrastructure. Cement is currently created using the dry method rather than the wet method, which necessitates an understanding of the significance of this industry's influence on the environment and human health and an attempt to develop remedies. Cement bypass dust is an odorless substance that exists in the form of particles or powder and ranges in color from white to grey. Wet dust has a pH of 12 to 14, indicating that it is intensely alkaline. The solubility of bypass dust is minimal^10,11^.

Synthesized zeolites are required for wider market applications worldwide. This can aid in avoiding the environmental dangers associated with waste materials. The definition of zeolites is crystalline aluminosilicates. Chemically, they have the general formula M_2_/nO.Al_2_O_3_.ySiO_2_.w H_2_O, where y is between 2 and 200, n is the valence of the cation, and w represents the water contained in the cavities of the Zeolite^12^.

This research aimed to identify fungi associated with rot bulbs of onion during the storage period and develop control using Cement and Zeolite materials, which have great alkalinity, and evaluate their efficiency against fungal growth of *Aspergillus* section *Nigri* attacking bulbs of onion after harvesting.

## Materials and methods

### Samples collection, isolation, and identification of fungi

Thirteen samples of preserved rotten onion bulbs were collected from marketplaces in Qena, Egypt. The day after collection, microbiological quality testing, and natural mycobiota identification were conducted ^13^. The dilution-plate method was used to survey the mycobiota associated with onion bulbs as stated by^14^. One gram of the sample was cut, transferred to 100 ml sterile distilled water and shaken for 10 min. One ml of the solution was transferred into a sterile petri dish, followed by adding Potato Dextrose Agar (PDA) medium. Incubation was performed at 28 ºC for 1 week. The fungal colonies were purified on PDA. Morphological features and macro-and microscopic characterization were determined by culturing the isolated *Aspergillus* section *Nigri* on Malt Extract Agar (MEA) medium at 28 °C for 7 days.

### DNA isolation from *Aspergillus* section *Nigri* isolates

*Aspergillus* section *Nigri* isolates were cultured on PDA medium, for two days at 28 °C. DNA was extracted using the cetyl trimethyl ammonium bromide buffer (CTAB) procedure from harvested growing mycelium as described by Ferracin et al.^15^.

### PCR amplification, sequencing, and phylogenetic analysis

E1m4 (5P-TGRGGWGCWACWGTTATTACTA-3P) and rE2m4 (5P-GGWATAGMWSKTAAWAYAGCATA-3P) were the forward and reverse primers, used in the amplification of cytochrome *b* gene based on the amino acid sequence, the primers were previously designed from cytochrome *b* amino acids^16^. The PCR reaction was done in a tube containing a total volume of 20 µL: 10 µL of 2X Tag master (Jena Bioscience, Germany), 0.5 µL from each primer (20 pmol/µL), 1 µL of the strain DNA (100 ng/µL), and 8 µL PCR ddH_2_O. The PCR reaction mixture was run for one cycle at 94 °C for 5 min, followed by 30 cycles consisting of denaturation for 1 min at 94 °C, annealing for 1 min at 50 °C, and extension for 2 min at 72 °C and final extension at 72 °C for 10 min.

The quality of obtained PCR products was checked on agarose gels (1%) stained with ethidium bromide and photographed by UV light on gel documentation (USA).

Sequencing and sequence analysis of the cytochrome *b* amplicons were done, the PCR products were purified using a Qiagene PCR purification kit (Qiagene, USA) and submitted to be sequenced (in Macrogen company, Korea).

Chromas Lite software was used to edit the cytochrome *b* gene sequences, and Clustal W software was performed to align them. Using MEGA software 6.0, the neighbor-joining tree with bootstrap values was created^17^.

### Determination of ochratoxins A production by *A. niger* strains

According to Gabal et al.^18^, *Aspergillus* has an estimated ochratoxins potential. A conical flask containing 100 ml of YES broth with ingredients (g/L): sucrose 40 g and yeast extract 20 g was used to transfer an active culturing disc from a 7-day culture. After a two-week incubation period at 28 °C, the fungal filtrates were separated by filtration and then again filtered through glass fibre paper. The quantities of OTA were calculated in 10 ml that passed through an ochratoxins column (VICAM, Watertown, MA, USA) at 1–2 drops/s. The ochratoxins were eluted using 1.5 mL of ochratoxin elution solution after the columns had been washed twice with 10 mL of deionized water. The concentration of ochratoxins A was determined on a VICAM series-4 Fluorometer that had been recalibrated^19^.

### Survey of polyketide synthase gene (*pks*) in *A. niger* strains

Polyketide synthase gene (*pks*) in *A. niger *was detected. It is well known that a Polyketide Synthase Encoded by the Gene An15g07920 is involved in the biosynthesis of ochratoxins A in *Aspergillus niger*. So, to examine the fungi harbour this gene which is accordingly able to produce ochratoxins. Aopks1 5′-CAG ACC ATC GAC ACTGCA TGC-3′ and Aopks2 5′-CTG GCG TTC CAG TAC CATGAG-3′ primers were used to amplify the *pks* gene^20^. The reaction was carried out in a tube that included ten μL of Tag master 2X (Jena Bioscience, Germany), 0.5 μL of each primer (20 pmol/uL), one μL of template DNA (100ng/uL), and completed to 20 μL of deionized water. PCR condition was as follows: denaturation at 94 °C for five min, 30 cycles at 94 °C for one min, 58 °C for 1 min, 72 °C for one min, and final extension at 72 °C for 10 min. The resulting PCR product was scanned using the Gel Documentation & Analysis system (USA), stained with ethidium bromide, and tested on a 1.2% agarose gel.

### Characterization of Cement bypass dust and Zeolite

Cement bypass dust is environmentally harmful; therefore, getting rid of it and reusing it is preferable. The raw bypass dust was collected from a cement factory in Qift City (Qena governorate, Egypt). The Cement bypass waste was used without any modification or purification. The Cement bypass dust is characterized by a high concentration of alkalis, especially chlorides, K_2_O and SO_3_^21^.

El-Nahas et al.^22^ described the method used to synthesize the Zeolite sample. Aluminium wastes were converted into Zeolite during the procedure using a household microwave. Finally, the collected Zeolite materials were filtered, rinsed with deionized water, and dried at 110 °C overnight. Zeolites are aluminosilicate solids with a negatively charged honeycomb structure of micropores that allow molecules to be adsorbed to catalyze chemical processes and clean up the environment. They are essential to green chemistry because they reduce the need for organic solvents^23^.

### Fungal growth inhibition assay

Using a modified method of the fungal growth inhibition experiment reported by Fiori et al.^24^, the antifungal activity of Cement and Zeolite against *A. niger* was evaluated. The necessary concentration (as we tested different concentrations) 10 × 10^3^ µg/mL was achieved by combining the powdered Cement or Zeolite with the melted PDA, this test was done by duplicate plates and the mean of them was calculated. The fungal isolate was cultured on PDA medium for 7 days, and an 8 mm-diameter disc of the growth was placed on the surface of the treated medium, incubation was done at 28 °C for 7 days. PDA medium without treatment was utilized for the control. After one week, the colony diameter was measured, and the percentage of fungal growth that was inhibited in comparison to the control treatment was determined using the following formula:

$${\text{I}}\, = \,{\text{C}}\,{-}\,{\text{T}}/{\text{C}}\, \times\, 100$$

where I is the percentage inhibition, C is the radial growth in control, T is the radial growth in treatment (test).

### Management post-harvest bulb rots of onion

Cement and Zeolite powder were used to control the post-harvest healthy onions. The bulbs were cleaned and sterilized by ethyl alcohol (70%) for 1 min and dried by tissue under sterilized conditions. Thirteen bulbs for each treatment were covered by the tested powder, and sprayed by a concentrated spore suspension (1 ×10^8^ spores/mL) of the tested fungal isolates. Thirteen untreated onion bulbs served as controls, sprayed by the fungal suspension. Bulbs without treatment and fungal suspension were also used. Onion bulbs were incubated at 28 °C for eight days. The percentage of rotten fruits and their disease severity were determined. Pathogenicity rating was recorded as the mean of duplicate bulbs for each isolate of *A. niger*, Less virulent: < 50%; Moderate: 50–75%; Highly virulent: more than 75%^25^.

### Permission

All collected samples were transmitted to our lab in South Valley University after were complied with relevant institutional, national, and international guidelines and legislation and transferred safely to mycological analysis.

## Results

### Mycobiota of stored bulbs

All the collected samples (n = 13) of stored bulbs were positive for fungal contamination with a percentage of 100% of *Aspergillus* section *Nigri*. Thirteen isolates of them were chosen for further characterization.

### Macroscopic and microscopic examinations of *Aspergillus* section *Nigri*

**Figure 1:**
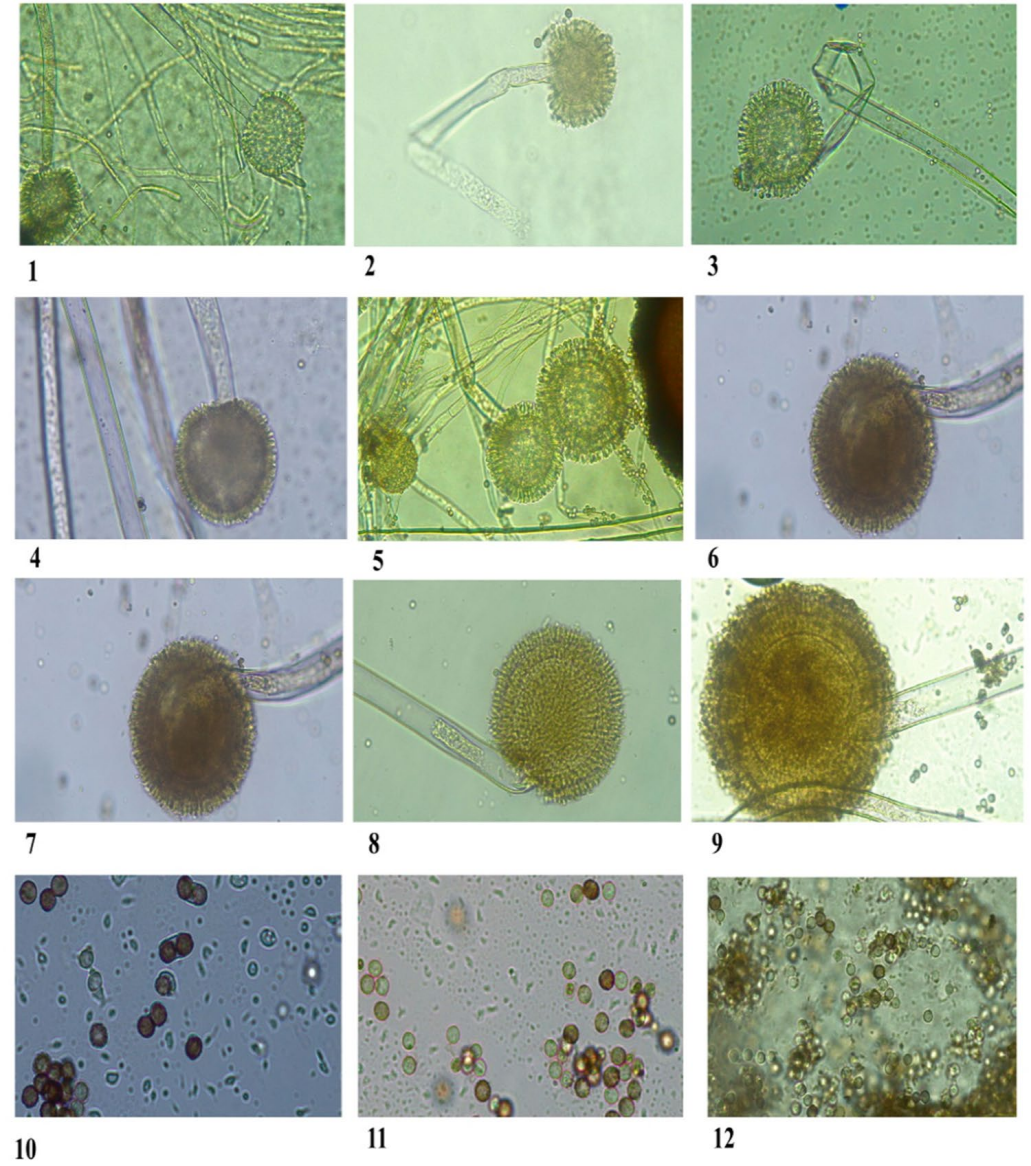
Microscopic examination of isolated *Aspergillus* section *Nigri*, obtained from rotten onion bulbs, collected from Qena Governorate, Egypt, on MEA medium at 28 °C for 7 days. From left to right: 1–9: radiate and loosely radiate, uni and biseriate conidial head with ×40, 10–13: Conidia with ×100.

**Figure 2:**
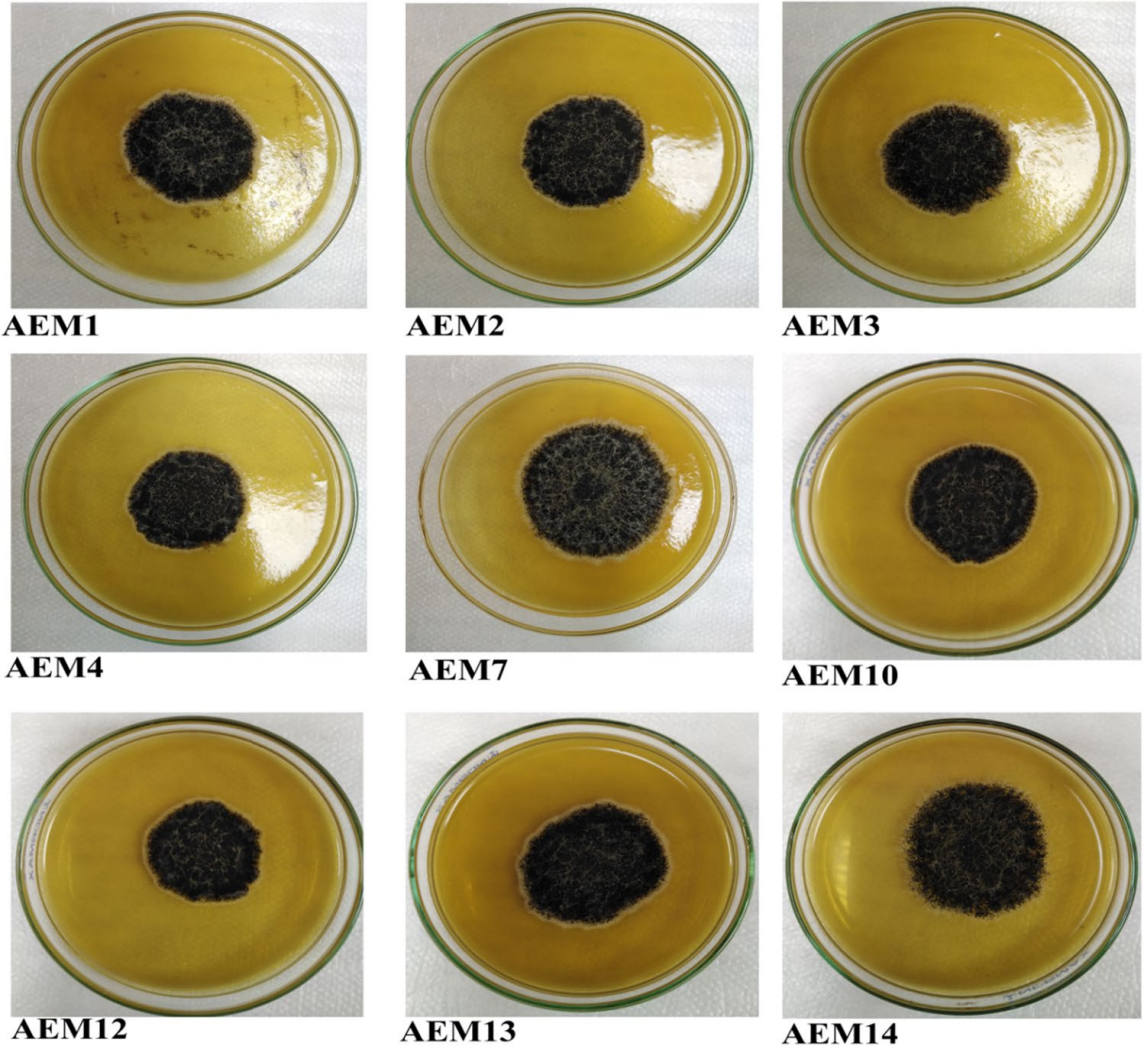
Colony colour, and texture of tested *Aspergillus* section *Nigri*, isolated from onion bulbs on MEA medium at 28 °C for 7 days.

Based on macroscopic and microscopic criteria, we identified the thirteen collected isolates as *Aspergillus* section *Nigri* in all onion samples. In all isolated strains, *Aspergillus* section *Nigri* strains were characterized macroscopically as a black colony with a narrow white border. Colony size was 29–50 mm. The colony texture was floccose, with white mycelium sparse on the colony. Microscopically: vesicle size was 33.958–82.736 µm, vesicle shape was sub-globose to globose, conidial head was radiate and loosely radiate; uni and biseriate, conidia shape and color were sub-globose to globose; smooth or finely-to-distinctly roughened; colorless to brownish color. Conidia size was 2.459–7.862 µm (Table 1 and Figs. 1, 2).

**Table 1:** Macro and microscopic criteria of *Aspergillus* section *Nigri* on MEA medium at 28 °C for 7 days.

Morphological characteristics	Description
Colony colour	Black with narrow white edge
Colony diameter (mm)	29–50
Colony texture	Floccose with white mycelium sparse on the colony
Vesicle size (µm)	33.958–82.736
Vesicle shape	Sub-globose to globose
Conidial head	Radiate and loosely radiate, uni and biseriate
Conidia shape and colour	Sub-globose to globose, smooth or finely-to-distinctly roughened, colourless to brownish color
Conidia size (µm)	2.459–7.862

### Molecular identification of *Aspergillus* section *Nigri*

**Figure 3:**
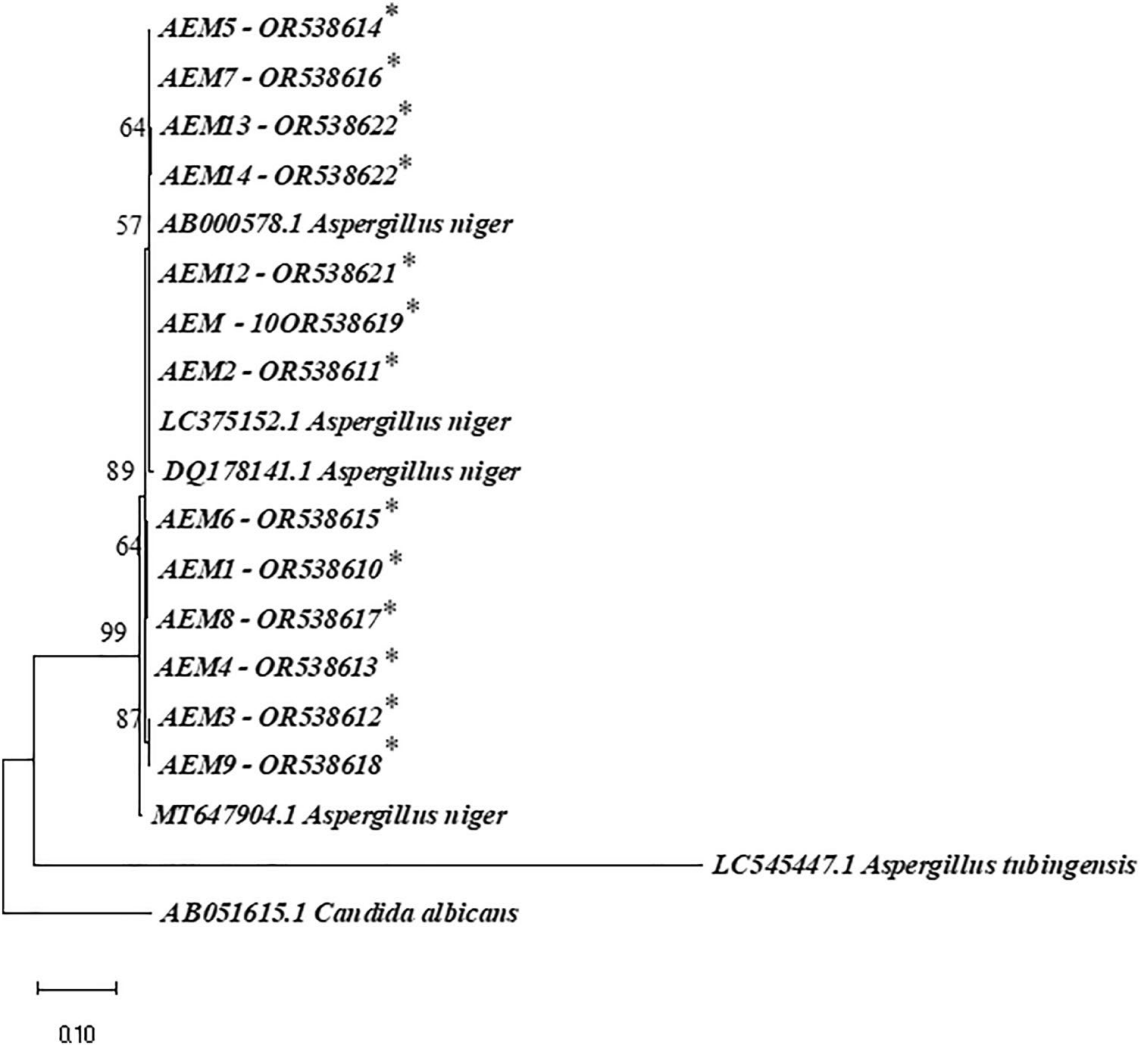
Phylogenetic tree reconstructed from the cytochrome *b* gene sequences of isolated *Aspergillus niger* aligned with the other sequences from *Aspergillus* section *Nigri* deposited in the NCBI database. *Candida albicans* was used as the outgroup. The numbers above branches indicate bootstrap values that were constructed after a run of 1000 replications.

Using the designed primer E1m-E2, the 437 bp fragments of the cytochrome *b* gene were amplified from all thirteen strains of *Aspergillus* section *Nigri*. Our strains of *Aspergillus* section *Nigri* showed similarity with 99–100% to the species of *A. niger* deposited at the Genbank. Based on the phylogenetic tree, the strains of *Aspergillus niger* were grouped with other *A. niger* sequences LC375152.1, AB000578.1, DQ178141.1, MT647904.1, achieved from the GenBank database with a bootstrap more than 80% (Fig. 3). The accession numbers of these isolates were inserted in the phylogeny tree.

### Detection of ochratoxins A efficiency and *pks* gene of *Aspergillus niger*

Thirteen isolates of *A. niger* were subjected to ochratoxins A analysis. Three isolates were detected to produce ochratoxins A, and their production ranged from 1.5 to 15 ppm. Isolate of AEM7 was the highest producer of ochratoxins A. *Pks* gene was not detected in all tested isolates, although three isolates exhibited the production of ochratoxins A (Table 2).

**Table 2:** Codes, accession numbers, ochratoxins A exhibited by *A. niger* isolates, and surveying of *PKS* gene.

Isolate code	Accession numbers	OTA levels (ppm)	*pks* gene
AEM1	OR538610	0	0
AEM2	OR538611	1.5	0
AEM3	OR538612	0	0
AEM4	OR538613	0	0
AEM5	OR538614	0	0
AEM6	OR538615	0	0
AEM7	OR538616	15	0
AEM8	OR538617	3.7	0
AEM9	OR538618	0	0
AEM10	OR538619	0	0
AEM12	OR538621	0	0
AEM13	OR538622	0	0
AEM14	OR538622	0	0

### Characterizations of the synthetic Cement bypass dust and Zeolite

To determine the basic components presented in the disposal of Cement bypass dust and Zeolite, the X-ray Powder Diffraction Analysis (XRD) was studied at a 2θ range between 10° and 70°.

**Figure 4:**
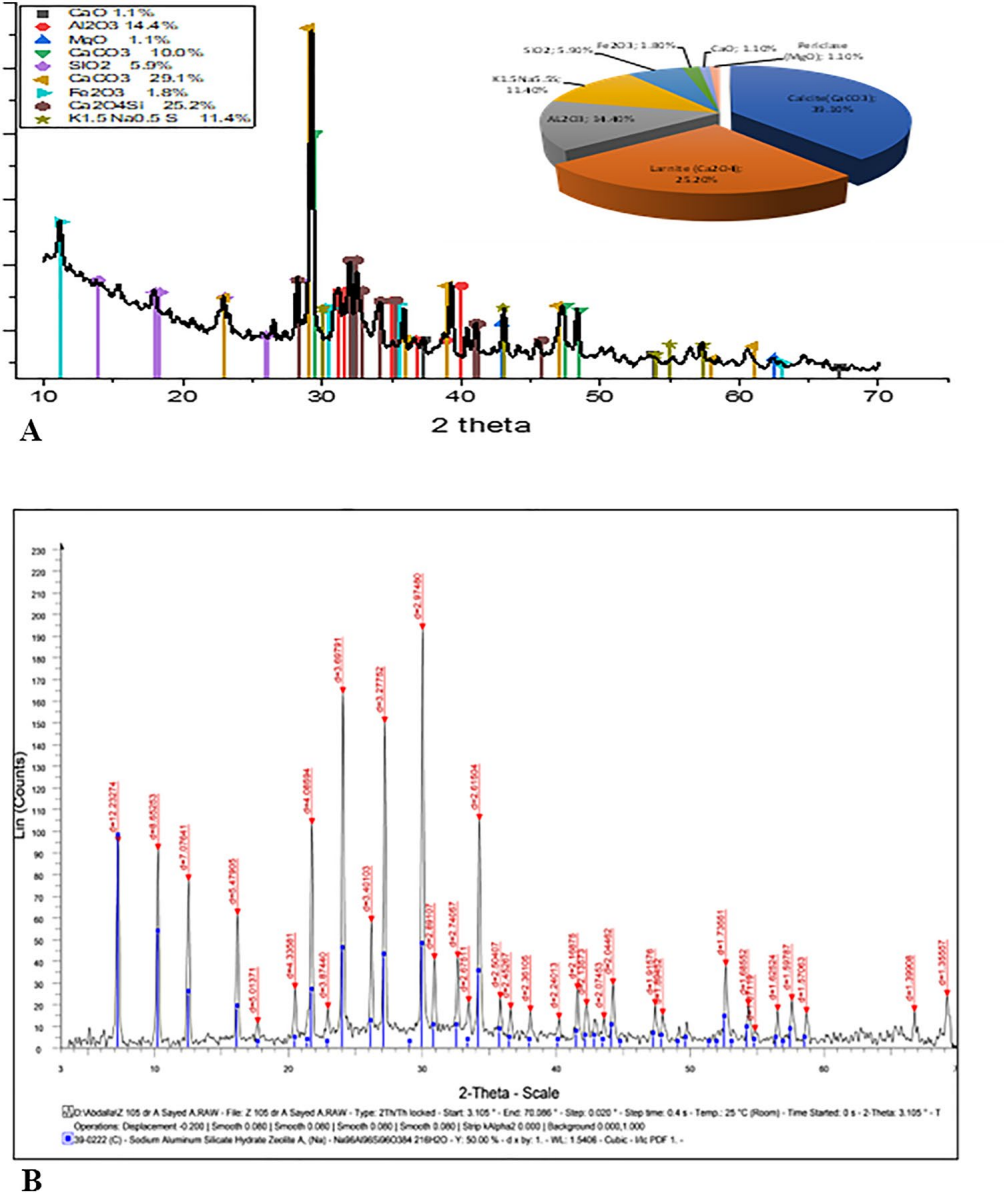
XRD pattern of the Cement waste and Zeolite.

As shown in Fig. 4, the results showed that several compounds were present, with calcite CaCO_3_ being the most common component percentage in bypass dust (39.1%), followed by Larnite Ca_2_SiO_4_ (25.2%) and Al_2_O_3_ (14.4%).

Zeolite A showed the characteristic peaks at 2θ values: 7.2°, 10.3°, 12.6°, 16.2°, 21.8°, 24°, 26.2°, 27.2°, 30°, 30.9°, 32.6°, 33.4° and 34.3° at diffraction lined: 12.3, 8.7, 7.09,5.49, 4.09, 3.7, 3.4, 3.28, 2.98, 2.89, 2.74, 2.67 and 2.62 respectively.

According to the Scherrer equation, the produced Zeolite was in the nanoscale range reached 21.7 nm, and had a cubic crystal structure with strong XRD peaks.

EDX (Energy Dispersive X-ray Spectroscopy) analysis provides the elemental composition of various constituent elements in the Cement waste materials. Ca contributed to 39.5% of the elemental composition. Si, Al, Na, Fe, and Cl had low percentages.

**Figure 5:**
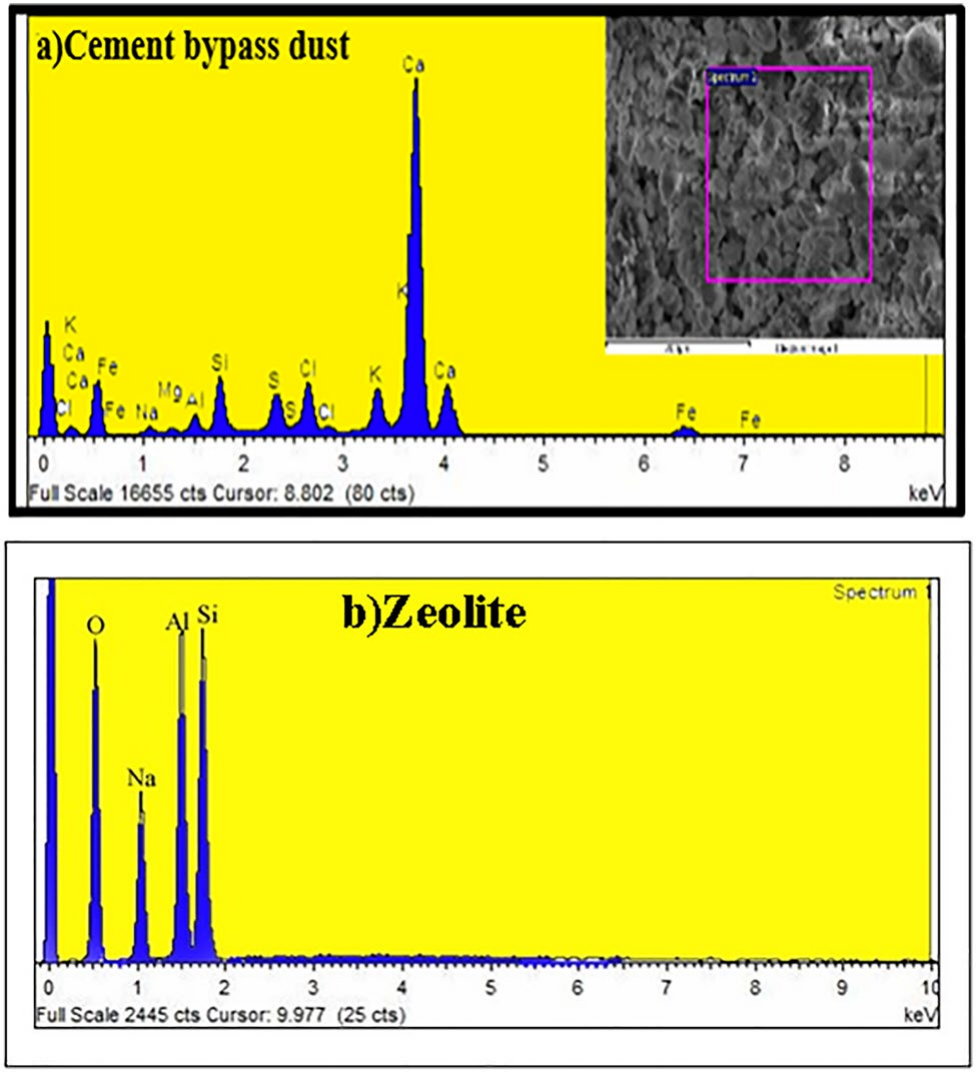
EDX spectrum of the Cement waste and Zeolite.

EDX analysis confirmed the elemental composition and constituent of tested types of Zeolite. Na, Al, Si, and O elements were observed in the tested sample. That confirms the purity composite phase with no impurities in Zeolite samples Fig. 5.

### Antifungal activity of safely waste Cement and Zeolite

**Figure 6:**
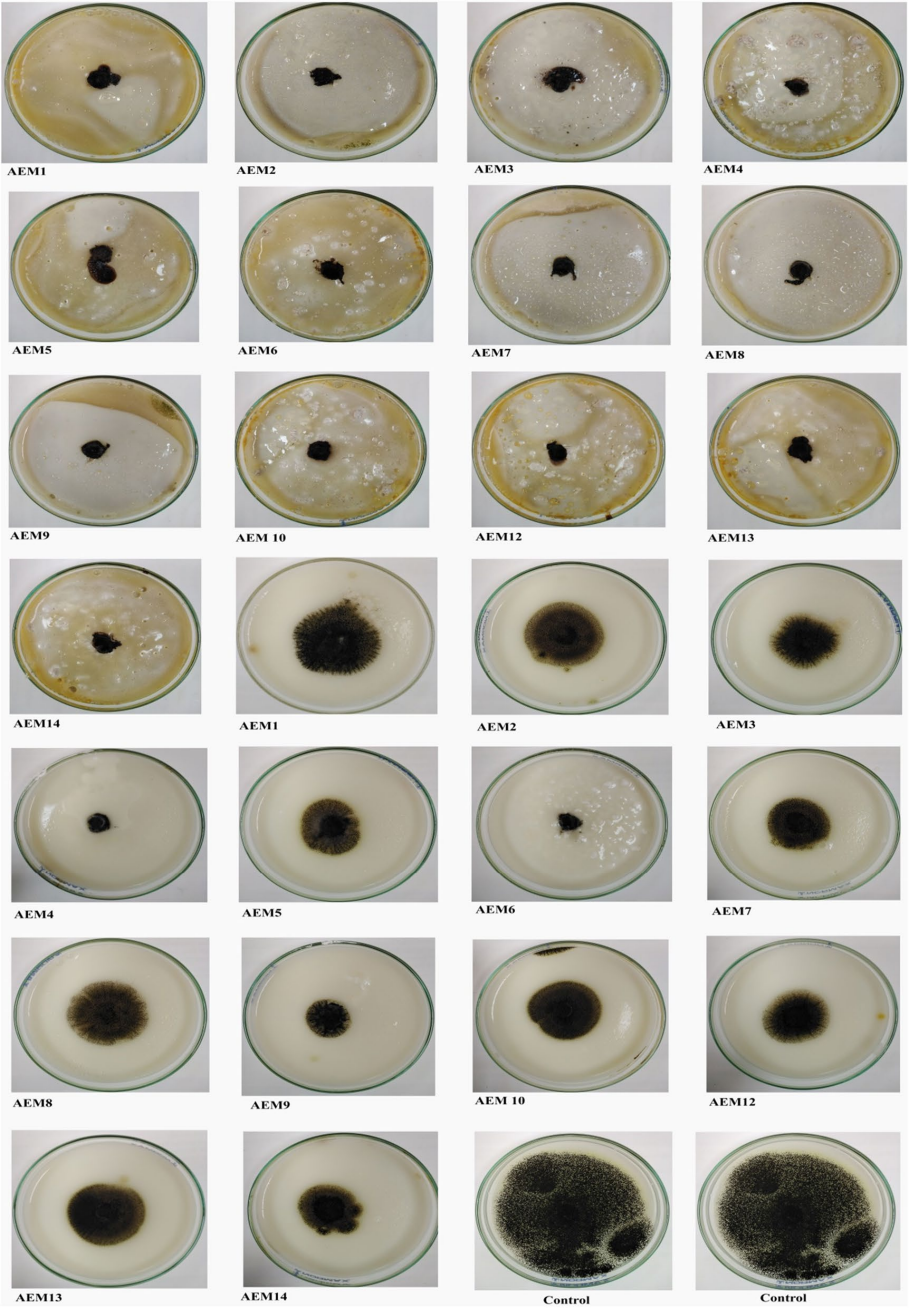
Antifungal activity of Cement and Zeolite on the growth of *Aspergillus niger* isolates on Potato Dextrose Agar medium at 28 °C for 7 days. From left to right: 1-13 plates treated with Cement, 14-26 plates treated with Zeolite, plates no. 27 and 28 were control.

The two wastes inhibited the growth of collected isolates with different ranges. Waste of Cement exhibited maximum percentage growth inhibition more than Zeolite against the tested isolates. Waste of Cement showed the highest percentage of growth inhibition against AEM5 isolate at 86.7% and the lowest percentage of growth inhibition against AEM1 at 74.7%. In the case of Zeolite, the highest percentage of growth inhibition was observed in isolate AEM4 at 85.3%, and the low percentage was recorded in isolates AEM1 and AEM 10 at 49.3% (Fig. 6 and Table 3).

**Table 3:** Effect of Cement and Zeolite on the fungal growth causing post-harvest rot of onion.

Isolate code	Treatments				
	Control (cm)	Cement (cm)	Percentage inhibition Cement (%)	Zeolite (cm)	Percentage inhibition Zeolite (%)
AEM1	7.5	1.9	74.7	3.8	49.3
AEM2	7.5	1.3	82.7	3.6	52
AEM3	7.5	1.25	83.3	3	60
AEM4	7.5	1.15	84.7	1.1	85.3
AEM5	7.5	1	86.7	2.9	61.3
AEM6	7.5	1.45	80.7	1.2	84
AEM7	7.5	1.6	78.7	2.9	61.3
AEM8	7.5	1.65	78	3.5	53.3
AEM9	7.5	1.25	83.3	2	73.3
AEM 10	7.5	1.15	84.7	3.8	49.3
AEM12	7.5	1.25	83.3	3.1	58.7
AEM13	7.5	1.25	83.3	3.5	53.3
AEM14	7.5	1.35	82	2.95	60.7

### Protective effects of Cement and Zeolite on post-harvest bulbs rot disease of onion

**Figure 7:**
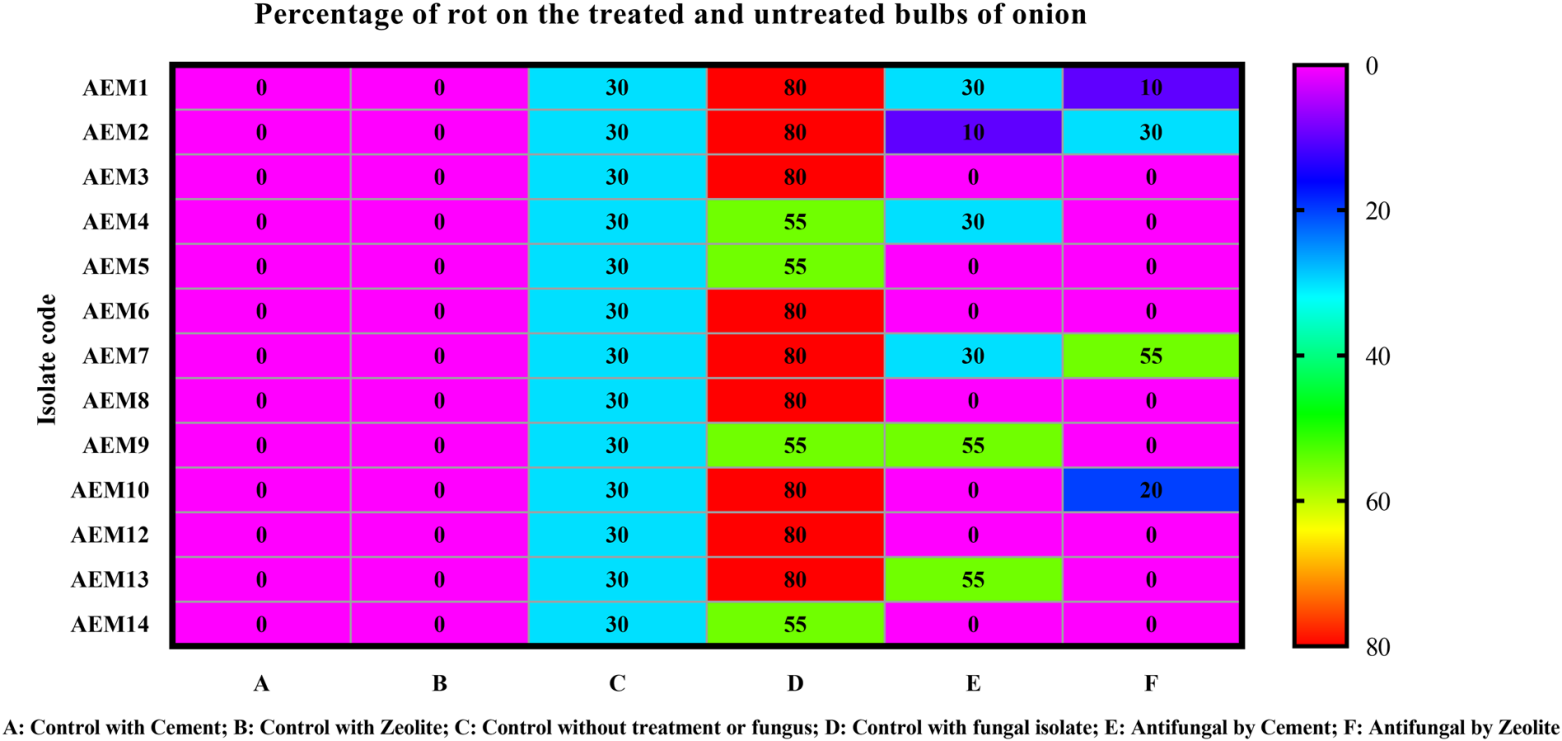
Percentage of rot after inoculation of onion bulbs with isolated *Aspergillus niger* isolates, after storage period at 28 °C for 7 days.

**Figure 8:**
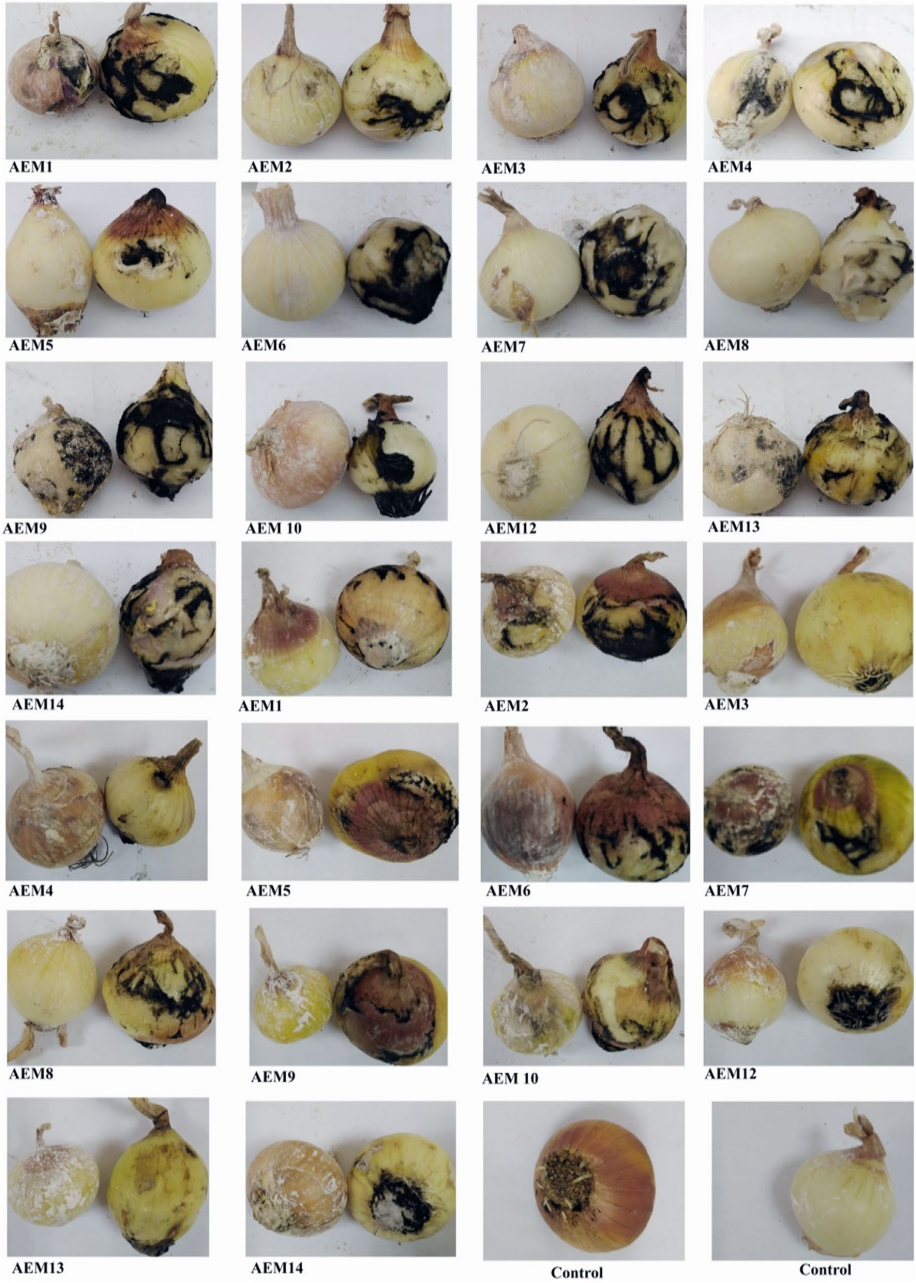
Protective effect of Cement and Zeolite on post-harvest bulbs rot disease. Onion bulbs were kept in storage at 28 °C for 8 days. Each image representing: onion covered with treatment at the left and inoculated onion at the right. From left to right: 1–13 Cement treatment, 14–26 Zeolite treatment, 27 and 28 were control covered with Cement and Zeolite.

The results revealed non-growth or slight to moderate growth of *A. niger* in treated bulbs. The two treatments significantly reduced bulbs rot disease of onion than untreated bulbs. Seven strains of *A. niger* were completely suppressed by Cement treatment, and they were AEM3, AEM5, AEM6, AEM8, AEM 10, AEM12, AEM14. Nine isolates were inhibited in the growth when covered with Zeolite treatment; they were AEM3, AEM4, AEM5, AEM6, AEM8, AEM9, AEM12, AEM13, AEM14 (Figs. 7 and 8).

## Discussion

Thirteen isolates of *Aspergillus* section *Nigri* were isolated from the samples (n = 13) of stored onions from markets in Qena. All the tested onions showed contamination by black Aspergilli; which were characterized by morphological, physiological and molecular criteria.

Under hot and humid conditions, black mold (*A. niger*) is a serious contaminant of onion bulbs^5,26^. Earlier mycological studies confirmed the fungus's ability to spread via seeds, soil, and the air^27^. *Aspergillus niger* was demonstrated to be transmitted from them to developing seedlings and onion sets^28^. When the aerial leaves die, the fungus either enters through the neck, wounded roots, or scales^29^. However, harvesting onions before the leaves are fully dried results in more storage rots and lowers the market value of the bulbs^30^.

Varga et al.^31^ examined six samples of onion for the presence of black aspergilli. They were able to effectively isolate black aspergilli from each sample examined. According to Gherbawy et al.^32^, 37 onion samples from the Taif region contained black aspergilli. *Aspergillus* section *Nigri* was expected to be isolated from 98 percent of the onion samples. Multiplex PCR allowed for the differentiation of *A. niger*/*A. welwitschiae* strains from other *Aspergillus* section *Nigri* species. According to Massi et al.^6^, *A. niger*/*A. welwitschiae* was significantly more prevalent than other *A. niger* aggregate species among 500 randomly selected fungal isolates.

The *Aspergillus niger* "aggregate" consists of a variety of morphologically similar species of *Aspergillus* section *Nigri*, including *A. niger*, *A. brasiliensis*, *A. tubingensis*, *A. welwitschiae,* and *A. luchuensis*^8^. Morphological and molecular identification were used to confirm the characterization of black Aspergilli in this study. The analysis of the phylogeny, classification, and identification of black Aspergilli can be done using the cytochrome *b* sequences. The first study using the mt cytochrome *b* gene in the phylogenetic analysis of fungi was Wang et al.^16^. Due to the discovery of species-specific sequences in the cytochrome *b* gene, *Aspergillus* strains can be distinguished according to the species level. For *Aspergillus* strains, conventional morphological identification is challenging or unavailable, examining the sequences is a helpful tool for precise identification and taxonomy. Information about evolutionary relationships among interspecies to intraspecies level can be found using the cytochrome *b* gene.

This study found that three isolates out of thirteen exhibited ochratoxins A production, and none of them showed the tested *PKS* gene. Genes involved in mycotoxin production are frequently grouped, co-regulated, and co-expressed in fungal genomes. Genes encoding one or more types of "core" enzymes required for producing the metabolite backbone structure are typically found in these gene clusters^33^. They may contain genes for regulatory proteins, hydrolases, oxidases, methylases, and other enzymes that are involved in adjusting the backbone structure to make the final metabolite, such as polyketide synthases (*PKS*) and non-ribosomal peptide synthetases (NRPS) genes ^34^.

Ferracin et al.^15^ examined 119 *A. niger* strains for the presence or absence of the *pks *gene as well as their ability to produce OTA. The presence of the *pks* locus in the *A. niger*/*A. welwitschiae* genome was positively correlated with the capacity to produce OTA, as demonstrated by the detection of the *pks* gene in every strain that produced OTA (n = 31) but not in any non-producing strains (n = 88). Our investigation did not find the *pks* gene in all strains tested, even though three of the examined isolates were positive producers. This may have been caused by a lack of amplification product or a low *pks* gene concentration or the production was correlated to *pks* gene region not studied here. *A. carbonarius* has demonstrated the ability to be toxic, as demonstrated by the culture plug method^35^, but its toxigenic (*pks*) gene was not amplified. Numerous investigations have demonstrated that the polyketide synthase (*pks*) and nonribosomal peptide synthetase (NRPS) genes influence the OTA production pathways in *Penicillium* and *Aspergillus* species^36^. A class of multifunctional proteins that includes both *PKS*s and NRPSs is involved in the synthesis of numerous secondary metabolites with a broad variety of biological functions. Amino acids and carboxylic acid moieties are the starting points for the biosynthesis of a large range of naturally occurring microbiological products. These hybrid NRPS-PKS systems, which involve direct interactions between NRPS and *PKS *modules, may accelerate the production of these mixed peptide-polyketide natural products, or they may work separately to build the peptide-polyketide backbone^37^.

This study characterization of Cement waste and Zeolite agreed with Salem et al.^38^. The presence of calcium hydroxide in CEM Cement is responsible for its effective antifungal activity, as shown by the research of Ayatollahi et al.^39^, who found that it inhibited the growth of *Candida albicans* except in the first few days. When CEM Cement's calcium hydroxide comes into contact with a medium, it breaks down into Ca^++^ and OH, which raises the pH. Inhibition of cell membrane enzymes occurs at alkalinities above nine^40^, meaning that such conditions can inhibit cellular activity.

When tested against *Candida albicans*, it was shown that Zeolite-Cu^2+^ and Zeolite-Zn^2+^ displayed a higher zone of inhibition. According to Cardoso et al.^41^, Zeolites are nanoporous alumina silicates in a framework with cations that display ion-exchange characteristics with metal ions, making them potential antimicrobial materials. According to Ozogul et al.^42^, Zeolite can be used as a natural addition to stop various biogenic amines, like cadaverine (CAD) and putrescine (PUT), from being formed by Gram-negative bacteria.

In conclusion, storing the bulbs in clean stores and covering them with Cement and Zeolite is an eco-friendly and safe alternative to synthetic fungicides against post-harvest rot of onion bulbs, it will most probably prolong the storage life of the bulbs against *Aspergillus* section *Nigri* that causes post-harvest rot.

## Data Availability

All data generated or analyzed during this study were included in this manuscript, the accession numbers of the sequences were deposited to the GenBank https://www.ncbi.nlm.nih.gov/nucleotide/, and inserted in the table.
